# Scraps

**Published:** 1892-09

**Authors:** 


### SCRAPS

PICKED UP BY THE ASSISTANT-EDITOR.

---

**Medical Philology** (III).—The Harleian MS. of the *Promptorium* has

ALBEREY, *vel* alebrey. *Alebrodium, fictum est.*

Pynson's edition printed in 1499 read "Albry." Mr. Way's note is:—

> "Alebery for a sicke man, *chaudeau*," PALSG.; which Cotgrave renders, caudle, warm broth.

*Aleberry* is found as a later spelling, and this led some etymologists astray. For instance, Dr. Johnson says the word is from *ale* and *berry*. But, as may be seen from the *New English Dictionary*, early forms were *alebre* and *alebrue*, and the latter part of the word is connected with the well-known *brewis*, which appearing with many variations has no etymological alliance with *brew*, and meant a kind of broth with small pieces either of meat or bread in it.

An aleberry was "a beverage made by boiling ale with spice and sugar and sops of bread in it." The quotations given in Nares's *Glossary* show that it was considered to be of nutrient virtue in cases where the appetite had failed.

**Clerical Licenses to Practise.**—The close connection of the Church with the early history of the healing art is well known. Licenses to practise were granted by Bishops or their Vicars-General, who maintained the right for a long period after the founding of the College of Physicians in 1518, and the incorporation of the Company of Barbers and Surgeons in 1540. The license was at times also given by other clerical authority. For two comparatively recent instances in this diocese, I am indebted to Mr. John Latimer, the author of *Annals of Bristol in the Nineteenth Century*, who has supplied me with the following memoranda. There is no date to the first of these, but as it was presented during the Chancellorship of Carew Reynell, it must have been between the years 1727 and 1743:

> "Petition of John Craighton, practitioner in physick and surgery, to the Dean and Chapter of Bristol, stating that he had practised for upwards of thirty years on board ships of war and in the army, and praying for a license to practise within the diocese, having no faculty to do so."
>
> "Faculty granted by William Cary, vicar-general of the diocese of Bristol, to John Coopey, permitting him to practise medicine in the city, deanery, and diocese. The faculty professes to be granted in consequence of Coopey's personal knowledge of medicine for divers years, and of the proof of his skill shown in his recent tract on the cure of diabetes. Dated 18 November, 1745."

**Professional Homes.**—The members of the medical profession in every city of a hundred thousand people should have their own home. They may be only able to rent a humble dwelling for such a purpose, but by all means rent until able to own, and there have a library, a reading and common sitting room, a dining room and kitchen. A fraternal spirit and union of hearts will there be knit together. Professional homes are promoters of peace and goodwill, and from them is sure to spring the benevolent sentiment that encourages the strong to care for and not step aside to crush out the life of the weak, and perhaps unfortunate. In the cultivation of this spirit and sentiment strong men are made stronger, and their lives more loveable. Their angular points are smoothed down until they are so polished as to on all occasions reflect the nobleness of soul that is within them, while the weak but deserving are held up and encouraged in practical ways.

A home is needed to bring about such desirable results. Once obtained, foster it with gifts as you would show your affection for a much loved child. Carry to it a book for its shelves, or the shelves to hold another man's book, a picture, a journal.—*Journal of the American Medical Association.*

**The Bath Water**—Added to Dr. Hall's book, from which I freely quoted in the *Journal* for September, 1891, are "Directions for fuch as drink the Bath-Water." These, collected by H. Stubbs, Physician at Warwick, were revised and approved by Sir Alexander Frasier, principal Physician to Charles II. Some extracts from these directions will be acceptable, more especially as Mr. H. W. Freeman does not refer to them in his interesting book, *The Thermal Baths of Bath.* A preliminary warning is given to practise moderation in all things when making use of mineral drinks. This moderation itself has perhaps no inconsiderable share in producing the good results attributed solely to the waters. "The Effect of the *Bath-Waters* being drunk, is, to *difcharge* the whole Body from all *Impurities* that are incorrigible; to *correct* thofe that are *capable of Amendment;* to *free* the *innate heat* or *Ferments* of each part which are oppreffed through any *furcharge*, or peccancy of Humors; and to *reftore* or *revive* thofe that otherwife *languifh*, or are fome way decayed. . . . . The Effects they have in reference to *Tranfpiration* infenfible, are particularly evinced by this obfervation of the Laundreffes in *Bath*, *That thofe which drink the faid Waters, do foul their Linnen more than other People do, or than themfelves do at other times, when yet it is equally worn.* . . . . To the moft beneficial operation of Medicaments, it is requifite that there be an univerfal εὔροια, that the Humors be *fluxile* and the Paffages *pervious:* The which is fo much the more to be regarded in refpect to the *Bath-Waters*, becaufe they do not as other Medicaments, operate after one *determinate manner*, but more *catholicly*. But more efpecially Obftructions of the *Mefentery* are to be regarded, becaufe the Waters are more particularly to pafs through thofe parts."

After a caution "that thefe Waters are not to be looked upon as being in themfelves an *entire Courfe of Phyfic*," two rules are laid down as to "the *Manner* of drinking thefe Waters, which the Generality of Perfons ought to follow."

"I. That they *begin with drinking of the Waters, and conclude* (if their condition permit) *with bathing.* . . . . to begin with a Courfe of *Bathing and Sweating*, and to terminate with a Courfe of *drinking the Waters*, is not only *irrational*, but *dangerous*."

"II. *That they begin to drink thefe Waters by degrees, proceeding from a fmaller quantity to a greater; and having arrived to the largeft Dofe, there to ftay feveral days; and then by the like degrees contract their draughts, until they defift.* The whole Courfe of drinking being thus finifhed in fourteen days, or twenty days at moft."

At some watering places there are persons prepared to risk the dangers arising from a breach of the first rule. A lady at a German Spa was describing her symptoms to a friend. Wishing to explain that she first visited the bath, and afterwards took her glass of mineral water at the spring, she said: "My dear, the treatment is very simple. You take your bath first, and drink the water afterwards."

It was suggested by Steevens that when, in Sonnet 153, Shakspere wrote:

"I fick withall the helpe of bath defired,
And thether hied a fad diftemperd gueft,
But found no cure,"

he was referring to the Bath waters. This will always remain uncertain. No editor has been found to print the word bath with a capital initial.

